# A Bilingual AI-Based Chatbot for Nutrition Education in a Food Is Medicine Intervention for High-Risk Pregnant Women: Design and Development Study

**DOI:** 10.2196/85292

**Published:** 2026-05-01

**Authors:** Lorena Macias-Navarro, Nalini Ranjit, Gregory W Bounds, Brendon A Providence, Yesmeena Shmaitelly, Naomi M Tice, Shreela V Sharma

**Affiliations:** 1 Center for Healthy Communities, UTHealth Houston School of Public Health at Houston, 1200 Pressler St, Houston, TX, 77054, United States, 1 713-500-0146; 2 Michael & Susan Dell Center for Healthy Living, Department of Health Promotion & Behavioral Sciences, UTHealth Houston at Austin, Houston, TX, United States; 3 Brighter Bites, Houston, TX, United States; 4 US Department of Transportation (USDOT), Volpe Center, Cambridge, MA, United States

**Keywords:** artificial intelligence, AI, chatbot, nutrition education, Food is Medicine, pregnancy, human-centered design

## Abstract

**Background:**

Conversational agents (artificial intelligence [AI]–based chatbots) offer a novel approach to health interventions by providing personalized, adaptive interactions that improve over time based on user engagement. In nutrition education, given the wide variation in knowledge, skills, and abilities across participants, AI-based chatbots have the potential to enhance accessibility, engagement, and behavior change. Food is Medicine (FIM) interventions, which aim to improve food security and diet quality among multicultural, at-risk populations, often face challenges related to sustained engagement and use.

**Objective:**

This paper describes the design, development, and iterative refinement of a bilingual AI-driven nutrition chatbot integrated into an FIM intervention for high-risk pregnant women receiving care at obstetric clinics in Houston, Texas.

**Methods:**

The chatbot was developed using an iterative process informed by behavioral theory, human-centered design (HCD), and plan-do-study-act (PDSA) quality improvement cycles. The conversational agent was embedded within an ongoing 2-arm randomized controlled trial (N=200) comparing standard FIM nutrition education to FIM plus AI-driven nutrition chatbot support. HCD activities took place prior to deployment and involved community advisory group members and implementation stakeholders. Postdeployment refinements were guided by 2 PDSA cycles and informal question-and-answer sessions conducted with intervention arm participants. Qualitative feedback was collected using structured scripts to identify facilitators of and barriers to chatbot engagement.

**Results:**

The chatbot was developed using the GPT-3.5 Turbo application programming interface. An initial prototype built in Python using Gradio enabled rapid testing but lacked flexibility for modifications. To improve scalability and logging capabilities, the system was rebuilt using PHP, HTML, JavaScript, and SQL. To further understand usage patterns, participants who interacted with the chatbot at least once or not at all (classified as low users; n=32) were engaged in question-and-answer sessions. Of these participants, all were female (32/32, 100%), 88% (28/32) identified as Hispanic or Latino, and 90% (29/32) preferred Spanish. Two PDSA cycles guided iterative refinements. Cycle 1 identified low initial engagement, whereas cycle 2 focused on improving content clarity and cultural relevance through physical reminder prompts. Qualitative findings identified key barriers to engagement, including high cooking self-efficacy with perceived lack of need for support, low technology self-efficacy, and low urgency due to competing priorities.

**Conclusions:**

Embedding a bilingual AI-driven nutrition chatbot within an FIM intervention was feasible and featured critical design and implementation considerations for engaging high-risk pregnant populations. Findings show the importance of HCD and iterative refinement to address engagement barriers. This work provides actionable guidance for integrating conversational agents into FIM programs, with implications for future evaluation of clinical outcomes, long-term engagement, and scalability.

## Introduction

### Background

Chatbots are defined as conversational agents powered by artificial intelligence (AI) designed to replicate human interaction via text, speech, and visual forms of communication, representing a novel solution to limitations regarding user experience [1,2]. Conversational agents can be designed to leverage machine learning and natural language processing to deliver personalized, interactive support to users, addressing their unique needs and preferences [1]. A recent meta-analysis highlights the effectiveness of chatbots, particularly text-based ones, in promoting lifestyle behaviors, including fruit and vegetable (F&V) consumption [3]. Moreover, these interventions have been shown to be effective across populations and age groups, reinforcing their potential for scalability and adaptability [3]. However, despite these promising findings, there remain gaps in the development, testing, and evaluation of conversational agent interventions designed specifically for high-risk, multicultural, low-income populations. These populations may face unique barriers when engaging with existing chatbots, including limited health or digital literacy, lack of linguistic accessibility, and reduced trust in automated health technologies that are not culturally or contextually tailored [1,2]. For example, some nutrition chatbots are designed for English-speaking users with high digital literacy and may not adequately address the needs or the realities of pregnant individuals navigating food access through Food is Medicine (FIM) programs [3].

Digital health innovations benefit from being grounded in human-centered design (HCD), a methodology that emphasizes iterative development through empathy, stakeholder involvement, and usability testing to ensure that tools are both acceptable and effective. In health care, HCD has been shown to improve user adoption by aligning interventions with the unique contexts and capabilities of patients and health care providers (such as a parent, nurse, or physician) [4,5]. However, even well-designed interventions often require refinement once deployed in real-world settings. To address this, the plan-do-study-act (PDSA) cycle is frequently used as a continuous quality improvement framework that supports rapid testing of targeted changes, measurement of impact, and structured adaptation [6]. Combining HCD during initial development with PDSA for ongoing evaluation creates a complementary approach that allows digital health tools such as conversational agents to evolve responsively to user feedback and use patterns over time.

One potential area for testing conversational agents is nutrition education to support interventions that promote diet quality. FIM interventions are a set of interventions that are integrated into the health care system and provide consistent access to fresh, healthy food, including produce prescriptions, medically tailored groceries, and medically tailored meals, to address the critical link between nutrition and health [7,8]. These interventions may also include nutrition education to support healthy habits.

### Objectives

To address the need to support FIM programs with culturally relevant and tailored nutrition information, this paper details the design of a bilingual, AI-driven nutrition conversational agent to support an FIM program for women with high-risk pregnancies using HCD and PDSA methods. This paper reports on the iterative refinement process and preliminary use patterns, specifically the development and refinement phases that shaped the conversational agent, *Flora*. The study design and evaluation of behavioral and clinical outcomes are reported elsewhere.

## Methods

The primary aim of this paper is to describe the HCD and iterative refinement of the conversational agent; details of the randomized controlled trial (RCT) are provided only to contextualize the implementation setting.

### Implementation Setting

The conversational agent, *Flora*, was developed as a key component of an ongoing 2-arm RCT to assess the implementation and effectiveness of conversational agents on improving use and consumption of F&V among high-risk pregnant women who receive care at Harris Health obstetric clinics in Houston, Texas. The RCT randomly assigned 200 women to the intervention and control groups with 1:1 allocation.

Eligibility criteria were as follows: (1) receipt of prenatal care at Harris Health obstetric clinics, (2) age between 18 and 44 years, (3) medically confirmed viable pregnancy of 20 weeks or less, (4) designation as high risk (high-risk criteria include age of ≥35 years, overweight or obesity [BMI≥25.0 kg/m^2^ at prepregnancy self-report], or prior history of pregnancy hypertension or gestational diabetes), (5) residence within the zip code food delivery radius, and (6) confirmed access to a smartphone capable of receiving SMS text messages.

### Study Design

This paper reports on the design, development, and iterative refinement of the chatbot embedded within a single, ongoing 2-arm RCT. HCD activities took place prior to trial deployment and involved community advisory group (CAG) members and implementation stakeholders who were not part of the RCT. Postdeployment PDSA cycles and informal question-and-answer (Q&A) sessions were conducted exclusively with participants randomized to the intervention arm to inform real-time refinements to the chatbot. Clinical and behavioral outcomes from the RCT will be reported separately.

### Ethical Considerations

Participants were required to be able to provide informed consent and communicate in either English or Spanish, the 2 languages supported by the chatbot. Informed consent was obtained verbally via phone call by credentialed community health workers prior to enrollment. Additionally, participants were required to use their own devices; smartphones and data plans were not provided by the study. Limited technology experience was not an exclusion criterion.

To support participation, all intervention participants received a personalized link to access the chatbot via SMS text message, an instructional video demonstrating how to use *Flora*, and follow-up phone support from a trained community health worker. Throughout the study, participants also received reminder SMS text messages and phone calls encouraging chatbot use. Participants were compensated for their time and participation with a total of US $50 in gift card incentives distributed over the course of the study.

To protect participant privacy and confidentiality, all data were deidentified for analysis and presentation. Audio files, transcripts, and analytic files were stored on University of Texas Health Science Center at Houston’s (UTHealth Houston) secure SharePoint within a restricted-access folder, with access to any identifiable information strictly limited to designated personnel in accordance with institutional privacy and security requirements.

This study was approved by the Committee for the Protection of Human Subjects at the UTHealth Houston (HSC-SPH-23-0734). The trial is registered with ClinicalTrials.gov (NCT07165990).

### Development and Refinement Phases of *Flora*, the AI-Driven Nutrition Conversational Agent

#### HCD Process

##### Overview

Prior to deployment, development of the conversational agent followed an HCD approach for interactive systems. We followed the International Organization for Standardization guidelines (ISO 9241-210:2019), which define a repeating HCD cycle with four activities: (1) understanding the context, (2) specifying user requirements, (3) generating design solutions, and (4) obtaining feedback from the end user community. HCD has been successfully applied to co-designing health chatbots in previous work [9,10].

##### Understanding the Context

###### Stakeholder and Participant Involvement

To understand the specific needs, preferences, and challenges of implementing a conversational agent into an FIM intervention, we engaged 2 interdisciplinary stakeholder groups to participate in the iterative process of cocreating the conversational agent, *Flora*. The first stakeholder group was a CAG with 10 to 15 current or former FIM program participants not related to the main study. The second group included clinical and implementation partners, such as members of Harris Health System and program implementers including Brighter Bites, Planet Harvest, and the research team from UTHealth Houston.

###### CAG Meetings

We conducted 4 CAG meetings throughout 2024. In these meetings, approximately 10 to 15 pregnant women currently or previously enrolled in FIM programs were invited to participate. The topics for discussion during these meetings included F&V consumption, experiences receiving produce boxes throughout their pregnancies, suggestions on how to improve produce deliveries, wants and needs with reference to nutrition education, and motivators for or barriers to eating healthy during pregnancy.

###### Clinical and Implementation Partners

The research team integrated input from the clinical and implementation partners and experts in behavioral science to facilitate implementation and enhance engagement. While developing the conversational agent, we had weekly meetings with Harris Health, Planet Harvest, and Brighter Bites to gain feedback and input. An expert in marketing at the Wharton School of the University of Pennsylvania, Dr Cait Lamberton, provided advice to support the development and refinement of strategies to encourage participants to adopt and consistently engage with the conversational agent. The goal was 2-fold: to familiarize participants with the technology and assess whether using the chatbot improved F&V consumption during pregnancy.

### Specify User Requirements

With feedback from our stakeholders, the next step was to co-design the conversational agent to enhance participants’ use of the FIM program (measured via self-report of F&V intake) by engaging in SMS text message conversations and delivering personalized nutrition information relevant and conducive to behavior change [3].

To achieve this, the conversational agent dialog system included both relational capacities to establish and maintain a relationship with the user and persuasive conversational capacity to modify their behavior [11]. We incorporated evidence-based behavior change techniques from social cognitive theory [12] and the health belief model [13]. We used constructs such as self-efficacy, perceived benefits, knowledge, and social support to motivate users to engage with the technology [14,15].

To engage the users and “humanize” the chatbot [16,17], we gave it the name *Flora*. This name was selected via a brainstorming session where our stakeholders suggested female names that could be pronounced in English and Spanish that would be relatable to the users, who were predominately Hispanic female individuals. By applying these behavioral strategies, we aimed to improve both adoption of and sustained engagement with the chatbot, ensuring meaningful evaluation of its impact on dietary outcomes.

### Generate Design Solutions

#### Initial Prototype

*Flora* was designed to interact with users seeking healthy nutrition resources and information about healthy pregnancies. It uses the GPT-3.5 Turbo model (OpenAI) for understanding and generating responses, and the medium to interact with the user is through SMS text messages. This process involved several iterative steps. Initially, the team familiarized themselves with the ChatGPT interface and application programming interface by deploying a Python-based chatbot using Gradio. While this solution facilitated rapid implementation, it proved challenging to modify. Specifically, the Python-Gradio prototype offered limited flexibility for iterative updates for conversation logic, multilingual content, and data logging, making it less suitable for ongoing refinement and real-world deployment within a clinical research setting.

#### Final Architecture

**Figure 1.**
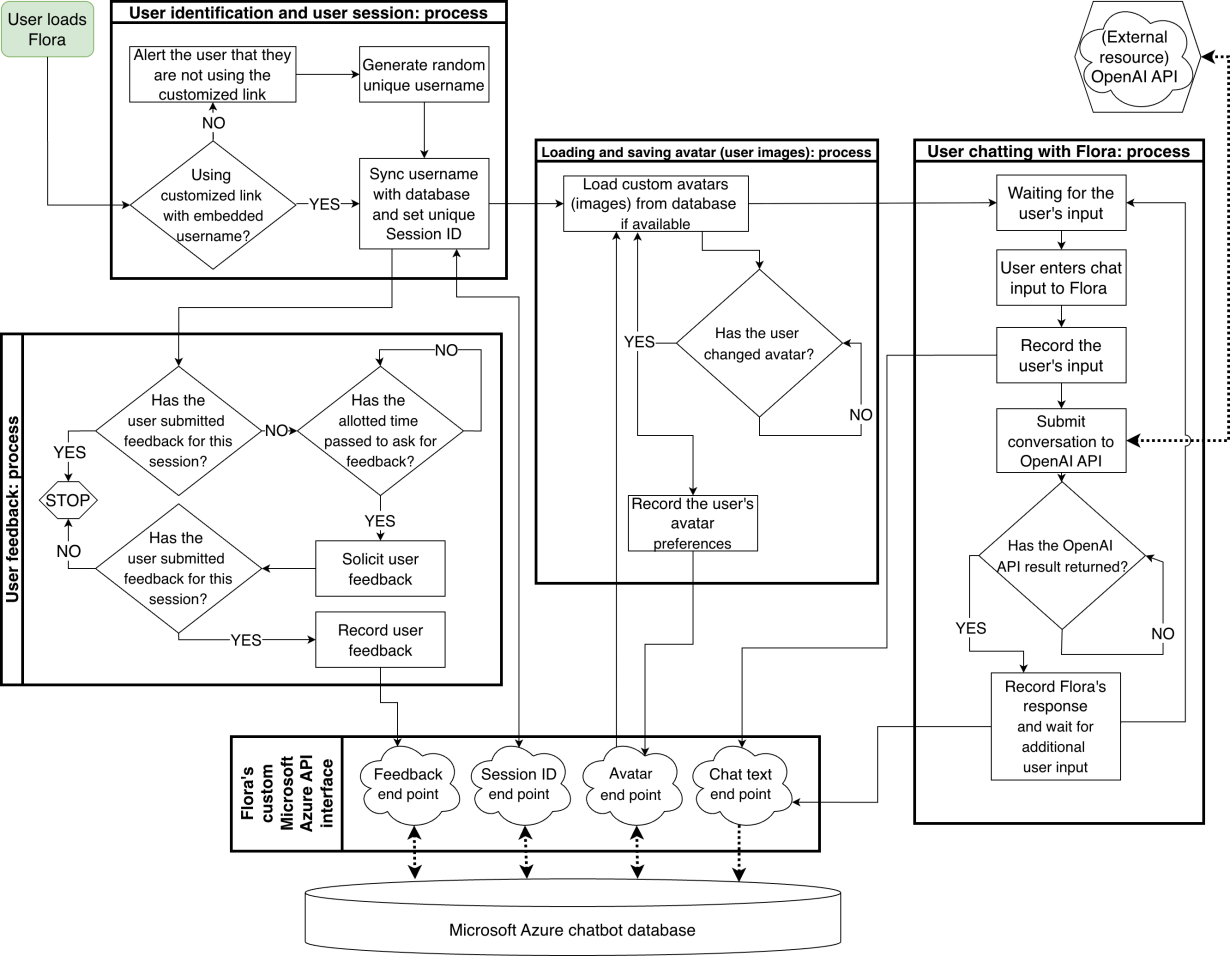
*Flora*’s architecture and workflow. API: application programming interface.

The team subsequently rebuilt the chatbot using technologies such as HTML, JavaScript, PHP, and SQL. The process began with research on PHP-based chatbot frameworks. A basic PHP chatbot was developed, which served as the foundation for integrating ChatGPT. This design allowed for effective separation of conversations and recording logs. Resources that informed development included guides on building PHP chatbots and integrating the OpenAI application programming interface. By leveraging the team’s expertise in PHP, they ensured that the chatbot was easy to modify, scalable, and efficient (Figure 1). The chatbot architecture included capacity to log interactions (time stamps, message content, language preferences, session IDs, and user ratings) to enable future evaluation.

Participants interacted with *Flora* through SMS text message–based, bilingual (English and Spanish) free-text queries using their personal mobile devices. They were encouraged to engage with the chatbot as often as desired during the intervention period, with typical sessions lasting a few minutes to obtain cooking tips or pregnancy-related nutrition advice. If *Flora* could not provide an appropriate answer, it redirected users with simple guidance (eg, suggestions for valid nutrition-related prompts). The research team reviewed use logs on a weekly basis to identify irrelevant outputs and inform iterative refinements. All interactions were logged in a secure, deidentified database that recorded time stamps, message content, language preference, and user ratings, with oversight from the UTHealth Houston study team to ensure quality and privacy.

#### Prompts and Personalization

**Figure 2.**
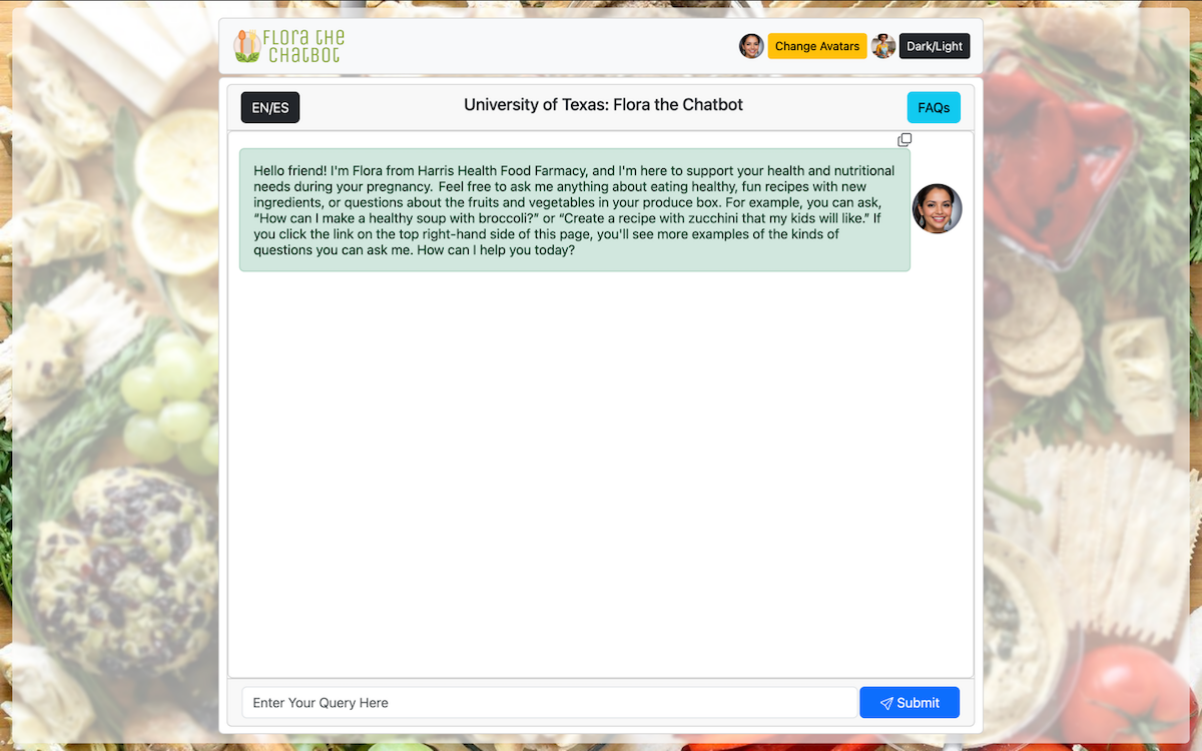
*Flora*’s interface.

**Figure 3.**
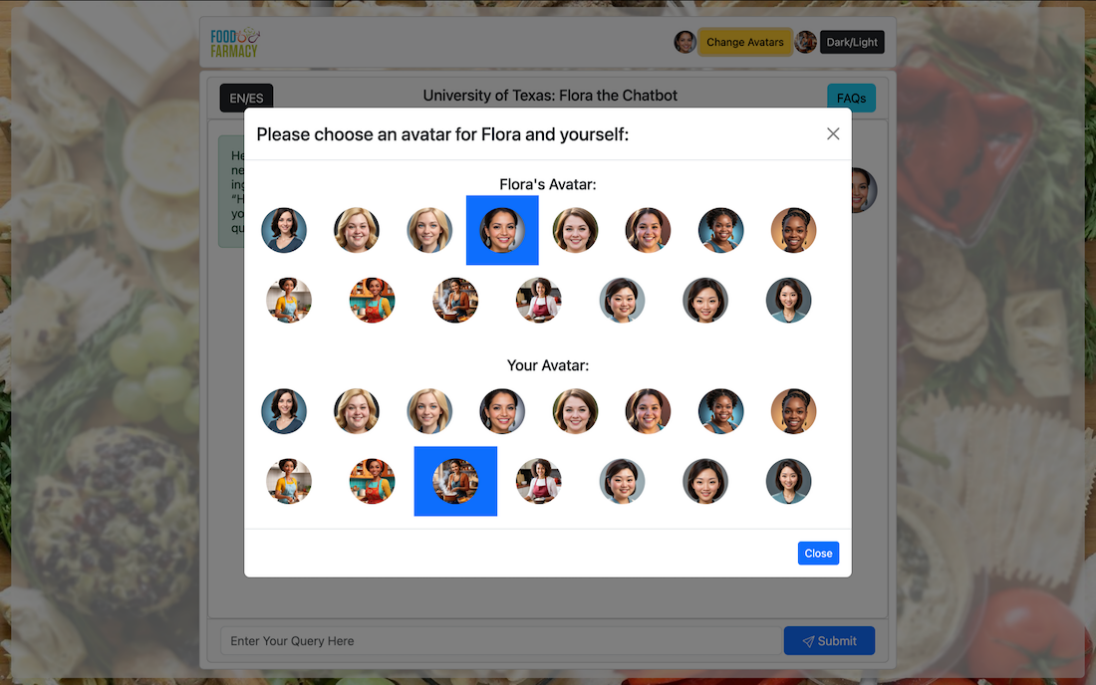
Options for users’ and *Flora*’s avatars.

The prompts used by the chatbot were iteratively developed to optimize its responses. Initially, prompts constrained the chatbot’s responses to recipes and nutrition-specific content. However, this approach limited its effectiveness in addressing valid user queries. Through trial and error, the team developed concise and structured prompts that framed the chatbot as a teacher guiding users toward nutrition-related and healthy pregnancy topics. *Flora*’s prompts support English and Spanish, with the ChatGPT engine recognizing and responding in the user’s input language (Figure 2). Moreover, we used an empathetic approach to communicating with the user in which the chatbot was prompted to respond with humanlike emotional interactions [16]. Personalization of *Flora* was further enhanced by allowing the user to assign *Flora* one of a wide variety of avatars differing by ethnicity and age (Figure 3).

### Feedback From the End User Community

To assess usability and refine the chatbot, we engaged both a CAG and program participants in iterative evaluation. CAG meetings were conducted as formative co-design sessions and focused on general perceptions, preferences, and design feedback rather than generating formal qualitative data. These sessions were recorded, and structured note-taking took place to capture key usability issues and suggestions. The CAG provided structured feedback on chatbot design features, including naming, tone, and cultural tailoring.

Moreover, informal usability testing and Q&A sessions with program participants were conducted to explore perceptions of accessibility, relevance, and overall experience. These sessions were designed to obtain rapid, implementation-focused feedback rather than to produce formal qualitative findings. Feedback from participants was independently reviewed and coded by 2 members of the research team to identify recurring barriers to and facilitators of chatbot use. Discrepancies were resolved through discussion until consensus was reached. Participant feedback was documented through structured notes and summarized to inform iterative refinements to chatbot content, prompts, and engagement strategies.

### Continuous Quality Improvement: PDSA Cycles

In addition to the structured HCD process, we anticipated the need for real-world refinements once *Flora* was deployed. To support this, we incorporated PDSA cycles as part of our continuous quality improvement approach. PDSA cycles, widely applied in implementation science [18], are designed to capture user experiences in practice and rapidly adapt interventions. Two PDSA cycles were planned for the postdeployment period between July 2024 and March 2025. The cycles were designed to identify usability barriers, inform iterative modifications, and document facilitators of engagement.

## Results

### Participant Context

The iterative refinement activities involved women enrolled in the FIM program who were largely Spanish preferring and of Hispanic or Latino ethnicity, reflecting the intended target population for the chatbot. Among participants engaged in follow-up Q&A sessions (n=32) who used the chatbot once or not at all, all were female (32/32, 100%); 88% (28/32) identified as Hispanic or Latino; 90% (29/32) preferred Spanish; and the mean age was 33 (SD 6.18) years, with a mean gestational age of 26.5 (SD 4) weeks.

### Qualitative Findings From Q&A Sessions

In February 2025, while the study was ongoing, the research team at UTHealth Houston conducted Q&A sessions with the FIM program participants via phone call. These Q&A sessions were conducted to contextualize early engagement patterns observed during deployment and inform interpretation of subsequent refinement activities.

To conduct these sessions, we developed a script that included questions regarding the following topics: confirming access to the chatbot, identifying challenges, providing technical support, encouragement to use *Flora*, and asking for feedback to improve their experience. Among participants contacted for Q&A sessions, themes related to low urgency and competing priorities were the most frequently reported, followed by confidence in cooking skills and low technology self-efficacy (Table 1). These findings are presented descriptively to characterize early barriers to engagement rather than to generate formal qualitative conclusions.

**Table 1.** Emerging themes and subthemes from participant feedback.

Themes	Subthemes	Participant quotes
Confidence in cooking skills reduced perceived need for chatbot support	High cooking self-efficacy Perceived lack of necessity	“I haven’t run out of ideas on what to cook. When I do, I’ll use it.” “I already know how to cook.”
Low technology self-efficacy	—^a^	“I receive many texts; I can’t find the link.” “My phone is very old, it’s hard for me to use the app.”
Low urgency and competing priorities	Low motivation for use Delayed use of the chatbot	“I got the link and then forgot about it.” “I haven’t used it because I’m busy, I’ll look at it later.”

a Not applicable.

### Iterative Refinement Results

#### Overview

**Figure 4.**
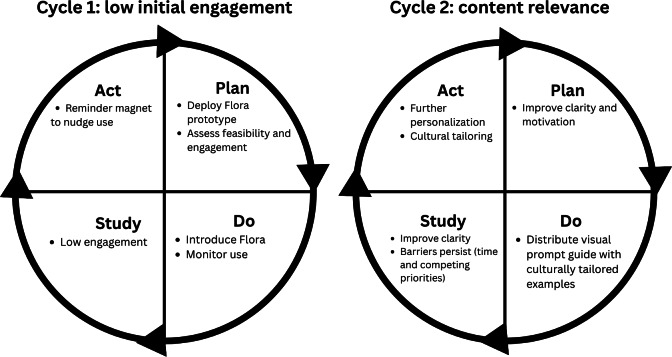
Plan-do-study-act cycles illustrating iterative refinements to *Flora* following deployment. Arrows indicate the iterative refinement process between cycles.

Following *Flora*’s deployment, 2 PDSA cycles were conducted to iteratively refine the chatbot in a real-world clinical context (Figure 4). These cycles were defined by sequential learning objectives rather than by isolating individual engagement components, reflecting pragmatic implementation conditions. Cycle 1 identified low initial engagement and tested a physical reminder strategy using a refrigerator magnet to encourage interaction with *Flora*. Building on these observations, cycle 2 focused on improving content clarity and cultural relevance through the introduction of a bilingual visual prompt guide.

#### Cycle 1: Low Initial Engagement

The first cycle revealed low participant engagement despite technical feasibility. Use logs showed limited log-ins (70/100, 70% of participants did not engage with *Flora*). Among those who used *Flora* at least once, the interaction sessions were brief (2 queries or less), suggesting that many participants did not return after initial exposure. Early feedback indicated that participants were uncertain about how best to interact with *Flora,* which limited its perceived usefulness.

**Figure 5.**
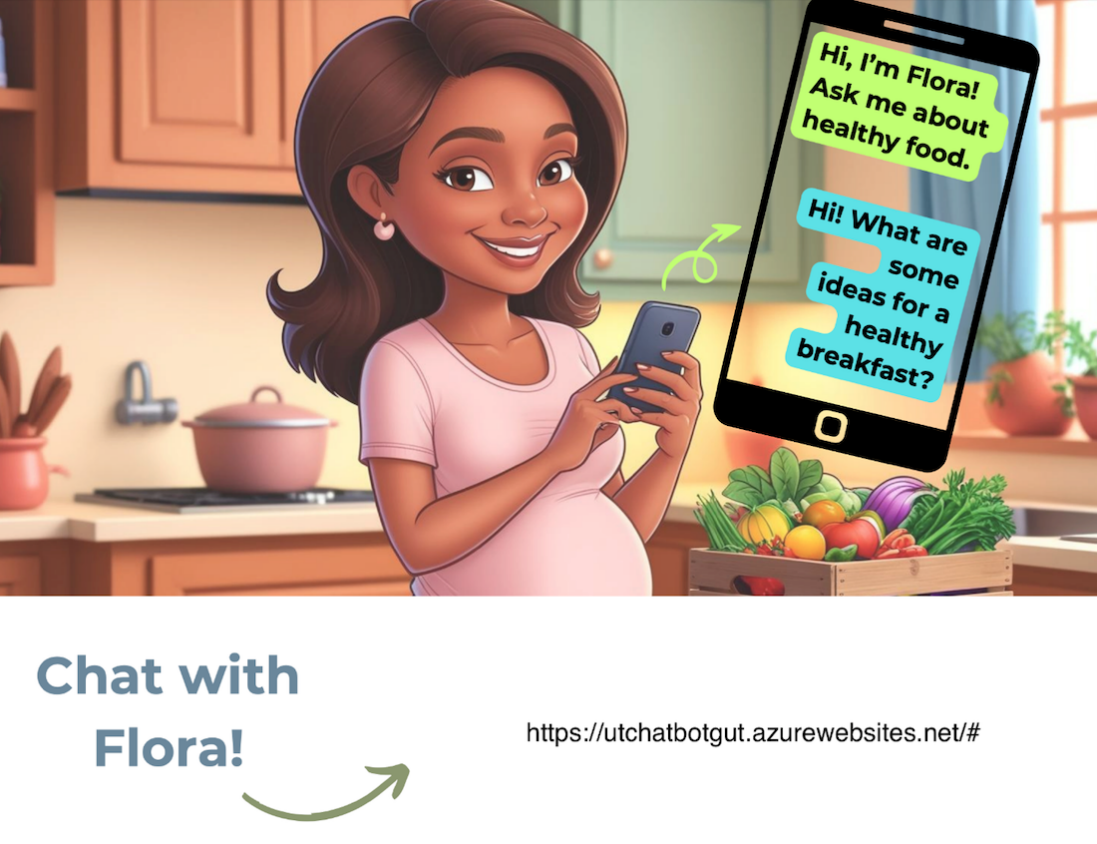
Refrigerator magnet design to nudge *Flora*’s use.

To address these challenges, the research team designed a refrigerator magnet that was sent to the participants’ home addresses as a tangible cue to encourage interaction with *Flora* (Figure 5). Following distribution of the magnet, use logs did not indicate a sustained increase in engagement, suggesting that a physical reminder alone was insufficient to overcome competing priorities or increase the perception of usefulness.

#### Cycle 2: Content Relevance and User Perceptions

**Figure 6.**
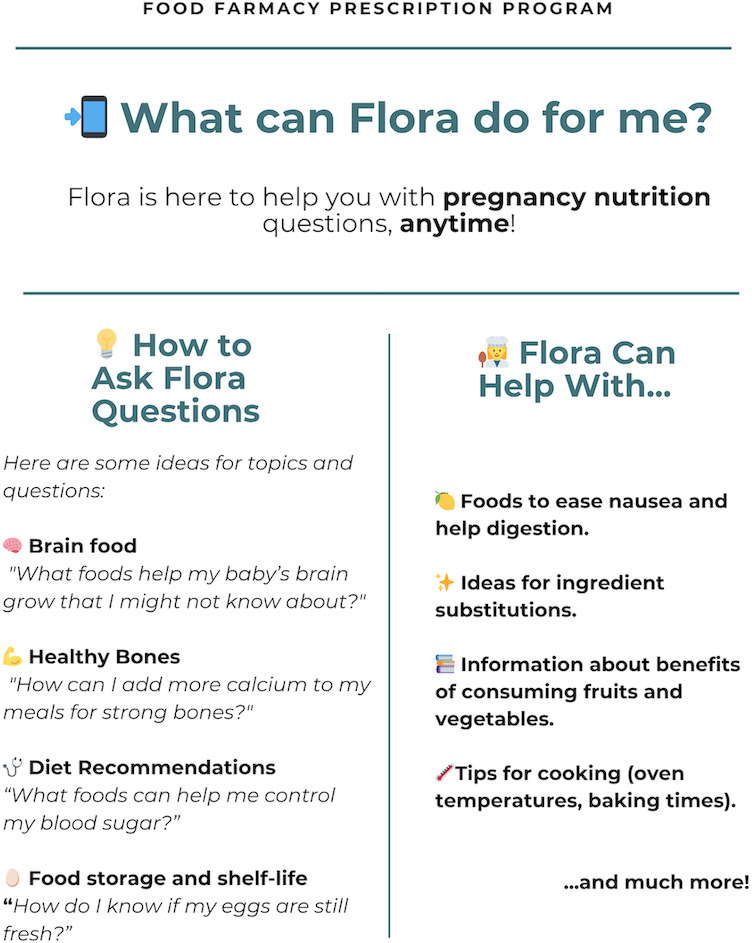
Prompt guide with tailored nutrition topics.

In the second PDSA cycle, the team implemented a visual prompt nutrition topic guide featuring examples of questions to ask *Flora*. The guide was developed in English and Spanish and was mailed to the participants’ homes (Figure 6) to clarify how to interact with *Flora* and reinforce engagement through tailored nutrition and pregnancy examples. Despite these efforts, engagement barriers persisted, particularly related to competing priorities and limited time. These findings highlighted the need for further personalization and cultural tailoring to sustain use.

While the PDSA cycles featured practical adjustments to improve engagement, they did not fully explain why participants continued to face barriers. To explore these underlying factors, in-depth qualitative semistructured interviews with participants are planned and will be reported in a subsequent paper.

## Discussion

### Principal Findings

The RCT this study was based on was designed to evaluate the feasibility and preliminary effectiveness of 2 nutrition education strategies associated with an FIM intervention among low-income, high-risk pregnant women in a clinical setting. To our knowledge, this is the first RCT to compare standard FIM nutrition education with an enhanced strategy that integrated an AI-driven nutrition chatbot. This approach allows for a rigorous assessment of health and behavioral outcomes as well as participant engagement and satisfaction with this technology.

The feedback from participants shows the promise and challenges of implementing a conversational agent within an FIM program. The emergent themes and subthemes provide valuable insights into barriers to adoption, such as low motivation or perceived lack of necessity, as well as facilitators, including openness to digital support. These findings point to several concrete opportunities for further tailoring conversational agent–based nutrition education within FIM programs. For example, for participants who reported high confidence in cooking skills and a perceived lack of need for chatbot support, future iterations could emphasize more advanced, novel, or pregnancy-specific nutrition content, such as food safety during pregnancy or creative uses of F&V with different spices or cooking techniques. For participants with low technology self-efficacy, engagement may be improved by simplifying access through alternative modes such as SMS text messaging–based interactions sent proactively from the chatbot rather than passively expecting the user to interact with the technology. Additionally, more frequent phone-based touchpoints could be useful to troubleshoot use issues. Finally, for participants facing competing priorities and limited time, the chatbot could be adapted to deliver concise, time-efficient interactions or embedded into existing routines, such as prenatal appointment reminders or produce delivery notifications. These strategies show how conversational agents can be tailored to different user profiles to enhance relevance, accessibility, and engagement.

Within the broader literature, personalized nutrition education remains limited in several FIM programs, and studies of AI-supported nutrition tools often lack rigorous comparative designs or adequate attention to language and cultural context. For example, reviews demonstrate that, while AI chatbots are growing in health promotion, there is a lack of rigorous RCT evidence demonstrating their effectiveness in modifying and maintaining dietary behaviors for weight management or chronic disease prevention in adults [19]. Additionally, literature on personalized nutrition education increasingly shows how AI-driven tools can enhance engagement when they are culturally and linguistically adapted [20]. This study addresses these gaps by implementing a bilingual, culturally adaptable AI chatbot nested within an RCT framework aimed at high-risk pregnant women participating in an FIM program. Grounded in behavior change theory, specifically social cognitive theory and the health belief model, this intervention integrates personalization, language choice, and interactive technology into a longer-duration evaluation framework [12,13].

Finally, this work contributes to the literature on clinic-based implementation strategies. As highlighted in recent research [21], successful program adoption depends on integration into existing workflows and staff practices. Demonstrating the feasibility and health impacts of an FIM program enhanced with AI technology has important implications for health policy. If effective, this model could inform broader adoption of conversational agents to support nutrition education and preventive care among vulnerable populations.

### Strengths and Limitations

This study has several strengths. The use of an RCT is a strength because it allows us to compare 2 different nutrition education strategies in a high-risk population of pregnant women. The evaluation data will be used to assess factors associated with implementation and adoption of these strategies. In addition, strong interinstitutional collaboration among the community, academia, and health care providers was critical to inform the design, implementation, and evaluation of implementation success.

This paper focuses on design, implementation, and iterative refinement rather than formal qualitative or engagement outcome analyses. The qualitative findings are presented descriptively to inform usability and implementation improvements and should not be interpreted as stand-alone qualitative results. A key limitation of this study is that *Flora* was built on a general-purpose language model (GPT-3.5), which may generate biased or incomplete responses.

To mitigate technology-related risks during the study, the research team implemented ongoing monitoring and quality assurance procedures. Chatbot interaction logs were reviewed on a weekly basis by the study team to identify potentially inaccurate, inappropriate, or unclear responses. Throughout the study, no major data safety issues were identified. However, system instructions were iteratively refined to improve accuracy, clarity, and alignment with evidence-based nutrition guidance. This process allowed for real-time oversight while preserving the scalability advantages of an AI-driven conversational agent in a clinical setting.

Emerging guidance on the use of large language models in health care emphasizes the importance of accuracy, transparency, and human oversight [22,23]. Future iterations of conversational agents such as *Flora* could further mitigate risks by incorporating model fine-tuning on verified, evidence-based nutrition content or using retrieval-augmented generation approaches that constrain responses to curated clinical or nutrition knowledge sources [24]. These strategies have been proposed as effective approaches to enhance reliability and reduce the likelihood of inaccurate outputs when deploying large language models in health-related contexts [25].

Another study limitation is that the web page–based implementation relied on participant-initiated engagement, which may have contributed to low overall use. Finally, reliance on self-reported measures may be subject to recall or social desirability bias; however, objective chatbot use data and process evaluation measures were included to provide additional context for interpretation and future research.

### Conclusions

This study demonstrates the feasibility of integrating a bilingual AI-based chatbot into an FIM program among high-risk pregnant individuals in a clinical setting. The iterative design and implementation identified engagement barriers, including competing priorities, technology self-efficacy, and perceived relevance while assessing the value of the human-centered approach and continuous quality improvement throughout the program. Lessons learned can inform the optimization of this program and provide guidance for future FIM programs seeking to incorporate conversational agents as scalable, culturally responsive nutrition education tools.

## Abbreviations

AI: artificial intelligence
CAG: community advisory group
F&V: fruit and vegetable
FIM: Food is Medicine
HCD: human-centered design
PDSA: plan-do-study-act
Q&A: question-and-answer
RCT: randomized controlled trial
UTHealth Houston: University of Texas Health Science Center at Houston
